# The Minimally Invasive Effect of Breast Approach Endoscopic Thyroidectomy: An Expert's Experience

**DOI:** 10.1155/2010/459143

**Published:** 2010-08-17

**Authors:** Wei Zhang, Zhi-guo Jiang, Dao-zhen Jiang, Xiang-min Zheng, Hong-liang Shen, Cheng-xiang Shan, Sheng Liu, Ming Qiu

**Affiliations:** 18F Minimally Invasive Surgical Center, Changzheng Hospital, Second Military Medical University, Shanghai 200003, China

## Abstract

We evaluated the invasiveness of breast approach endoscopic thyroidectomy (BAET) carried out by surgeon very experienced in this procedure. Twenty-four patients who underwent BAET and 19 patients who underwent conventional thyroidectomy were the study population. Postoperative pain was assessed by a visual analog scale (VAS). The values 2, 12, and 24 h after surgery were significantly lower in the BAET group than those in the conventional group. Serum IL-6 and CRP levels were measured by an ELISA preoperatively and at 2, 12, 24 and 48 h after operation. Their values increased significantly after both procedures when compared to preoperative levels with significant differences between the two groups detected at the 24-hour and 48-hour time points. Subjective and objective evidence supported the notion that BAET could become a minimally invasive procedure if the surgeon gained sufficient experience.

## 1. Introduction

Pursuit of an esthetically pleasing scar after open thyroidectomy has led surgeons to carry out endoscopic thyroid surgery [1]. Hüscher et al. [2] described the first case in 1997. Ikeda et al. [3] and Ohgami et al. [4] further improved the cosmetic result by carrying out endoscopic thyroidectomy via an axillary and anterior breast approach in 2000. This scarless (in the neck) endoscopic thyroidectomy (SET) attracted widespread attention due to the very good cosmetic effect achieved. The disadvantage of SET is a much larger plane of tissue dissection [1, 5]. Some surgeons think that SET should be classified as “minimal access but maximally invasive” surgery [1].

SET is usually related to longer operation time and more postoperative pain [1, 5], but some surgeons believe that these factors could reduced with the accumulation of experience [1, 6]. The learning curve of SET is very long, and a surgeon has to carry out about 150 SETs before reaching an advanced level in terms of the skill, proficiency, and stability of the operation [7]. 

Until August 2006, >230 cases of SET (all were breast approach endoscopic thyroidectomy (BAET)) have been carried out in our center. We believe that evaluating the invasiveness of this innovative surgical procedure at this stage is rational.

Current evaluation of surgical invasiveness related to SET relies solely on assessment of postoperative pain, so subjective bias cannot be avoided [8, 9]. Surgery is a unique example of “planned trauma” not only leading to pain, but also evoking a metabolic and inflammatory response (acute-phase response) characterized by release of various proinflammatory cytokines and increased production of acute-phase proteins [10]. Interleukin-6 (IL-6) and C-reactive protein (CRP) are valuable indicators reflecting the inflammatory and acute phase response after laparoscopic surgery [11–13]. The goal of this research was to evaluate the invasiveness of BAET, carried out by a very experienced surgeon, with subjective and objective indicators. 

## 2. Patients and Methods

### 2.1. Ethical Approval of the Study Protocol

All patients were informed about the study protocol. Signed written consent for the investigation in accordance with the ethical guidelines of Changzheng Hospital was obtained.

### 2.2. Patients

A prospective nonrandomized comparative study was conducted between September 2006 and May 2007. Enrolled in this research were 43 consecutive patients diagnosed with benign thyroid nodules. Surgical procedure (BAET or open thyroidectomy) was determined according to the wishes of the patient. Twenty-four patients (22 females and 2 males; mean age, 39.00 ± 9.17 years) who received BAET were assigned to the “endoscopic group”, and the remaining 19 patients (13 females and 6 males; mean age, 48.26 ± 10.47 years) received conventional thyroidectomy and were assigned to the “conventional group”. Exclusion criteria were a history of neck surgery or radiotherapy, thyroiditis diagnosed by preoperative biochemistry or ultrasonography, liver dysfunction, and immune system diseases.

### 2.3. Thyroidectomy

The endoscopic surgical procedure is described elsewhere [7]. BAET was carried out by one surgeon (Ming Qiu). Briefly, BAET was done under general anesthesia with the patient being in the supine position. A 12–15 mm curved longitudinal incision was placed along the medial contour of the right breast for the camera port, and two 5 mm incisions were made in the circumareolar region for the working ports. A working space was created with application of a harmonic scalpel under endoscopic guidance. The dissection plane was strictly over the muscular fascia. The lesion was exposed after the cervical linea alba was divided longitudinally. Partial, subtotal, near-total or total lobectomy was done according to lesion characteristics [14].

Conventional thyroidectomy was done through a 6 cm transverse cervical incision. Subplatysmal flaps were freed superiorly to the thyroid cartilage and inferiorly to the suprasternal notch. Then the lesion was exposed, and thyroidectomy was carried out.

### 2.4. Subjective Assessment of Postoperative Pain

Postoperative pain was assessed by a visual analog scale (VAS) consisting of a 10 cm line with the words ‘‘no pain” on the left side as “0” and ‘‘worst pain imaginable” on the right side as “10” [15]. All patients were asked to report their pain 2, 12, 24, and 48 h after surgery using the VAS.

### 2.5. Preparation of Blood Samples and Assays

Blood samples were obtained three days before surgery and 2, 12, 24, and 48 h after surgery. They were centrifuged at 1,250 × g for 15 min to separate the serum and stored at −70°C until assay. The IL-6 concentration was measured with an enzyme-linked immunosorbent assay (ELISA) kit (R&D Systems, Minneapolis, MN, USA) according to manufacturer's instructions. Briefly, the system used a solid-phase monoclonal antibody and an enzyme-linked polyclonal antibody against human IL-6. All analyses and calibrations were done in duplicate using 100 *μ*L serum for each analysis. Absorbance was determined at 450 nm. CRP concentration was measured automatically by nephelometry (BN II, Dade Behring, Marburg, Germany) according to manufacturer's instructions. The sensitivity was 0.7 pg/mL for IL-6 and 0.175 mg/L for CRP.

### 2.6. Statistical Analyses

Patient age, tumor size, incision length, blood loss, duration of surgery, and postoperative pain score were expressed as means ± SEM. Differences in these parameters between the two groups were analyzed by Student's *t*-test. Differences in pathologic findings (adenoma/nodular goiter) between the two groups were analyzed by *χ* ^2^ test. Differences in the change in the level of IL-6 and CRP between the two groups were analyzed by nonparametric test. Probability was determined using two-sided statistical tests. *P* < .05 was considered significant.

## 3. Results

### 3.1. Surgical Outcomes

The mean age of patients in the endoscopic group was significantly younger than that of the conventional group. There was no significant difference between the two groups with respect to tumor size, duration of surgery, and pathological type of lesions. The composition of different surgical procedures (partial/sub-near total/total) did not reach significant level between groups (2/13/9 versus 3/7/9, *P* = .487).The volume of blood loss and the length of incision were significantly reduced in the endoscopic group compared with the conventional group (Table 1). 

Complications such as recurrent laryngeal nerve (RLN) palsy, hypocalcemia, hypercapnia, subcutaneous emphysema, and seroma were not observed in either group.

### 3.2. Postoperative Pain

Postoperative pain began to increase 2 h after the end of surgery, reached a peak 12 h after surgery, and decreased gradually in both groups (Table 2). According to the VAS, pain severity on the first day of surgery in the conventional group was significantly higher than that in the endoscopic group and was not significant 48 h after surgery in both groups.

### 3.3. Change in Serum Levels of IL-6 and CRP

**Figure 1:**
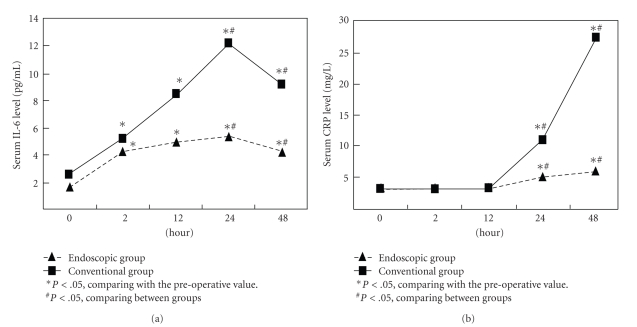
Time course of serum IL-6 and CRP levels among conventional and endoscopic groups before and after operation.

The serum level of IL-6 and CRP over time is depicted in Table 3. There was no significant difference in the baseline level of IL-6 and CRP between the groups (*P* > .05). Significant elevation of IL-6 was induced by endoscopic thyroidectomy and conventional thyroidectomy, reaching a summit 24 h after surgery whereas the CRP level increased later than did IL-6 and continued to increase even 48 h after surgery. Relatively slower and lower elevation of IL-6 and CRP levels was observed in the endoscopic group compared with that in the conventional group. There was no significant difference in the change of IL-6 and CRP levels 2 h and 12 h after surgery between the two groups, but they reached a significant level 24 h and 48 h after surgery (Table 3, Figure 1).

## 4. Discussion

Conventional thyroidectomy is a safe, effective, and well-tolerated method for treating various thyroid diseases. SET is an innovative procedure with extremely satisfactory cosmetic results, but we do not wish to assess the level of invasiveness of SET because many surgeons consider it to be a very technically challenging procedure [7–9]. We carried out this research only after we had reached an advanced stage of proficiency (carried out >230 cases) [7]. 

Minimally invasive surgery can be defined as the ability of the surgeon to carry out traditional surgical procedures in novel ways to minimize the trauma of surgical exposure [16]. IL-6 and CRP are the markers of the acute inflammatory response, and indicate the degree of surgical invasiveness [11, 17, 18]. Trends in the evolution of IL-6 and CRP over time in the present study were similar to those after abdominal surgery [11, 17], but the levels were lower. This supported the notion that thyroidectomy is not as extreme as abdominal surgery with respect to invasiveness [6]. As there was no significant difference between groups in composition of surgical procedures, the possibility of more invasiveness originated from more invasive procedure was ruled out. Based on our subjective and objective evidence, we concluded that BAET could be a minimally invasive approach for treating benign thyroid diseases. 

 Our initial experience showed that the operation time of BAET was significantly longer than that of open thyroidectomy [19], but some authors believe that the operation time could be reduced [1, 20]. Study of the learning curve confirmed that the operation time was significantly reduced and that the proficiency and stability of the operation reached an advanced level after the surgeon completed >150 cases of endoscopic thyroidectomy [7]. There was no significant difference in the duration of surgery between the two groups in the present study. 

 The dissection area in the anterior breast and neck is significantly larger than that of conventional surgery (data not shown), but does a large area of tissue dissection necessarily mean macroinvasiveness? The concept of endoscopic subcutaneous surgery had been introduced before, and it was also confirmed in animal models that dissection in this plane causes less trauma to the overlying and dissected tissues [21]. From an anatomic point of view, there is a potential space (the “fascia cleft”) between a distinct deep membranous layer of the superficial fascia and muscular fascia in the neck and the anterior breast region [22]. A working space was created along this layer in our series. We hypothesized that as long as the dissection was in the right surgical plane, invasiveness will not necessarily increase significantly even though the dissection area is relatively larger. This was confirmed by a study by Wang showing that endoscopic thyroidectomy neither enhanced the inflammatory response nor damaged human function despite extensive dissection of skin flaps [23].

 The reduced invasiveness of BAET could be attributed to its advantages over open surgery. The mean total length of the three incisions was only 2.9 cm in the current study. The shorter a single incision, the lesser disturbance to the integrity of anatomic structure it brought. In addition, endoscopic amplification of the visual field and the use of a harmonic scalpel enabled the resection to be more accurate and reduce the volume of hemorrhage [5, 20, 24]. Blood loss in the endoscopic group was significantly lower than that in the conventional group. The length of incision and total blood loss are important factors affecting surgical stress [25–27], so there is good reason to assume that BAET is advantageous over conventional thyroidectomy in reducing surgical trauma.

## 5. Conclusion

The present study demonstrated that BAET could be a minimally invasive procedure in expert hands. Attributable factors are shortened total length of incisions and reduced volume of blood loss. With accumulation of experience, the duration of surgery could be reduced to the level comparable with open surgery, and the invasiveness would not be enhanced despite the larger area of tissue dissection. 

## Figures and Tables

**Table 1:** Clinical data of the patients.

	Endoscopic group	Conventional group	*P*-value
Age (y)	39.00 ± 9.17	48.26 ± 10.47	.003
Tumor size in ultrasound (mm)	23.25 ± 8.84	27.78 ± 9.06	.056
Operation duration (min)	88.04 ± 28.73	90.05 ± 18.40	.793
Blood loss (ml)	17.88 ± 5.32	49.32 ± 10.93	.000
Length of incision (cm)	2.90 ± 0.28	6.16 ± 0.76	.000
Pathology (adenoma/nodular goiter)	23/1	17/2	.446

**Table 2:** VAS after thyroidectomy.

	Endoscopic group	Conventional group	*P*-value
2 h after operation	2.92 ± 0.50	3.78 ± 1.02	.00
12 h after operation	2.93 ± 0.81	5.42 ± 0.85	.00
24 h after operation	1.15 ± 0.44	2.03 ± 0.61	.00
48 h after operation	0.28 ± 0.41	0.43 ± 0.51	.31

All data are expressed in mean ± SD.

**Table 3:** Variation of serum IL-6 (pg/ml) and CRP (mg/L) levels after operation.

		Endoscopic group	Conventional group	*P*-value
IL-6	Before operation	1.65 (0.99–2.80)	2.66 (1.58–3.60)	
	2 h after operation	4.31 (2.47–6.43)*	5.21 (2.66–10.40)*	.420
	12 h after operation	4.98 (3.37–8.11)*	8.53 (4.70–13.32)*	.085
	24 h after operation	5.42 (2.95–8.39)*	12.2 (8.44–26.81)*	.003
	48 h after operation	4.32 (3.14–10.87)*	9.21 (4.62–17.99)*	.042

CRP	Before operation	3.19 (3.10–3.19)	3.21 (3.00–3.28)	
	2 h after operation	3.22 (3.08–3.30)	3.24 (3.11–3.33)	.949
	12 h after operation	3.25 (3.20–4.41)	3.28 (3.00–4.51)	.851
	24 h after operation	5.07 (3.19–8.03)*	11.10 (3.64–22.10)*	.030
	48 h after operation	5.92 (3.19–13.90)*	27.60 (15.60–66.00)^∗*^	.001

All data are expressed in median (P_25_–P_75_).

The difference in serum IL-6 and CRP levels between the two groups was analyzed with Wilcoxon rank sum test.

*P < .05, comparing with the preoperative value. The difference in postoperative serum IL-6 and CRP levels in both groups was analyzed with Friedman *M* test and *q* test.
